# Traffic Offenses, Court Charges, and Repeated Citations Among Teen Drivers With Traffic Violations

**DOI:** 10.55329/jwot3323

**Published:** 2025

**Authors:** Priyanka Sridharan, Rebecca J. McAdams, Saroj Bista, Dominique Rose, Corinne Peek-Asa, Jingzhen Yang

**Affiliations:** 1 Nationwide Children’s Hospital, the United States of America; 2 University of California, San Diego, the United States of America; 3 The Ohio State University, the United States of America

**Keywords:** recidivism, speeding, teen drivers, traffic offenses

## Abstract

Teen drivers with traffic violations are at high-risk for motor vehicle collisions (MVCs), yet this group is understudied. This study described traffic offenses, court charges (or dispositions), and repeated citations among teen drivers cited for traffic offenses, and examined factors associated with repeated citations. Deidentified traffic citation records for teen drivers ages 15–17, between January 2018 and May 2022, were obtained from Ohio’s Franklin County Juvenile Traffic Court in the United States. Frequencies for demographics, traffic offenses, court charges, and repeated citations (e.g., citation tickets) were generated and compared using Chi-square tests and linear mixed models. The final sample included 5 364 unique teen drivers (62.2% were male) with 6232 unique traffic citation tickets (13.9% were repeated citations). The most common traffic offense was speeding (28.5%), while the most frequent court charge was being an adjudicated juvenile traffic offender (38.2%). The likelihood of repeated citations increased by 3.61 (95% CI: 2.76–4.73) per additional year of driver’s age. Males (OR = 1.78; 95% CI: 1.43–2.21) and speeding offenses (OR = 2.29, 95% CI: 1.77–2.96) had higher odds of receiving a repeated citation than their respective counterparts. Males were more likely to be traffic offenders and receive repeated citations compared to females, consistent with established research on sex-related differences in driving behaviors. Speeding, a known risk factor for MVC-related fatalities, was associated with greater odds of repeated citations. Understanding the characteristics of traffic violations is crucial for developing targeted interventions, especially for males and speeding offenders, to promote safe driving and mitigate MVC risk.

## Background

Road traffic injuries and deaths continue to pose a significant global public health challenge (World Health Organization, 2023). In 2021 alone, an estimated 1.19 million road traffic fatalities occurred worldwide, with a global fatality rate of 15 per 100,000 individuals (World Health Organization, 2023). Traffic offenders of all ages are at an increased risk for motor vehicle collisions (MVCs) (Alver et al., 2014; Ayuso et al., 2010; Factor, 2014; Gebers & Peck, 2003; Goldenbeld et al., 2013; Summala et al., 2014), MVC-related hospitalizations, and deaths (Ayuso et al., 2010; Factor, 2014) than non-traffic offenders. Up to 73% of young adults report at least one traffic offense within seven years of receiving their driver’s license (Elliott et al., 2000), with a six-month re-offense rate as high as 56% (Ekeh et al., 2011; Manno et al., 2012). Fewer than half (about 45%) of novice drivers in California aged 16 and 17 years maintained a clean driving record during their first three years of unsupervised licensure (Chapman et al., 2014). This highlights the increased occurrence of traffic violations among young drivers, who continue to be disproportionately represented in both MVC-related injuries and fatalities (Insurance Institute for Highway Safety, 2021). Additionally, the recidivism rate for male teen drivers is 8 to 21 times higher than that of female drivers or drivers older than 18 (Carnegie et al., 2009).

Teen drivers with traffic violations represent a high-risk yet understudied population in the United States, where independent driving is permitted under the age of 18 (Insurance Institute for Highway Safety, 2015; McCartt et al., 2003). A recent scoping review found that existing research on individuals with traffic violations has focused on adult drivers, with limited attention to adolescents (Kaur et al., 2023). To address this gap, the current study aimed to 1) describe traffic offenses, court charges (or dispositions), and repeated traffic citations among Ohio teen drivers aged 15 to 17 cited for traffic violations, and 2) examine factors associated with repeated citations in this population. This research offers early insights into teen driver behavior, providing a foundation for future research and prevention efforts.

## Methods

### Study Data

We retrospectively analyzed deidentified records of traffic citation tickets issued to teen drivers aged 15 to 17 between 2 January 2018 and 31 May 2022. According to Ohio state law, juvenile drivers cited for traffic violations must deny or admit to it in juvenile traffic court. If the minor is found to be a juvenile traffic offender, the judge will enter a “disposition” which could result in a license suspension, paying court costs and fines, paying restitution to any victim of the offense, or probation or community control. If it is the juvenile’s second moving violation, there is a mandatory license suspension for a ninety-day period. Similarly, if the juvenile commits three or more moving violations, a mandatory one-year license suspension is imposed (Franklin County Juvenile Traffic Court). The data used for this study were obtained from the Franklin County Juvenile Traffic Court located in central Ohio, which manages all traffic citations and violations (i.e., convicted citations) for drivers under the age of 18.

### Case Selection

A total of 6783 unique traffic citation tickets were identified during the study period. Of those, 551 citation tickets were excluded due to: the driver’s age being under the legal licensure age (<15) or older (≥18) than when a full license is eligible (n=411), court hearing dates falling between 22 March 2020 and 6 July 2020 when the court was closed due to the COVID-19 lockdown (n=6), and various hearing date errors, such as a hearing date before the citation date (n=2), the same as the citation date (n=37), or over one year after the citation date (n=11) (see Figure 1). Additionally, tickets involving non-moving violations such as jaywalking (n=35), bicycle riding (n=15), and pedestrian involvement (n=34) were excluded. Since individuals could have more than one citation, the final sample included 6232 unique citation tickets corresponding to 5364 unique drivers.

### Variables and Measures

*Traffic offenses,* a categorical variable, was created based on types of traffic offenses, with up to eight offenses per citation ticket. The original data included 260 types of traffic offenses. We consolidated offenses with different wording but identical meaning into unified categories. This reduced the total to 51 distinct offense types (see Supplementary eTable1). We present the top 11 traffic offenses, which account for over 80% of all recorded offenses. They are as follows: speeding, no operator’s license (including no temporary permits), assured clear distance ahead (ACDA), failure to control, failure to yield, right of way, traffic control device, seatbelt violations, reckless operation, driving outside marked lanes, and failure to stop at a stop sign. All other offenses were categorized into “Other”. The variable was further coded for a *repeated citation* (i.e. a second or subsequent citation ticket issued to the same individual during the study period).

*Court charges*, a categorical variable, was created based on 14 original types of charges provided by the court. We excluded those without a decision or actual charge, such as “hold open,” “dismissal,” and “plea of no contest.” The 11 remaining charges were then consolidated into 8 categories: adjudicated juvenile traffic offender, dismissal – rule 29 (i.e., court dismissed a complaint based on rule 29 in the best interest of the child and the community) (The Supreme Court of Ohio), monetary fine (combining state offense and non-state offense fines), costs assessed, defensive driving course, suspended driver’s license, limited driving privileges, and community service.

*Demographics*, provided by the court, included a citation number, which was a unique identification code for the citation, age at citation, sex (male or female), race (White, Black, or Other), ethnicity (Hispanic or non-Hispanic), residential zip code of the individual, citation date, and court hearing date. Cases that had an initial court appearance also had a hearing date.

*Citation before vs. after COVID-19*, a dichotomous variable was created to indicate whether the citation date was before or after 9 March 2020, which was when Ohio declared a state of emergency for the COVID-19 pandemic.

### Statistical Analysis

The data were analyzed using SAS 9.4 (SAS Institute Inc., Cary, NC). Chi-square or Fisher’s Exact tests (for variables with cell counts <5) determined the association of traffic offenses (court charges) with age, sex, and race. Because a teen driver may have multiple traffic citations, generalized linear mixed models generated odds ratios (ORs) for repeated versus one-time citation within the study period, accounting for correlation between citations within an offender, and other relevant covariates. Backward selection with alpha = 0.05 cutoff was used to identify the most relevant variables for the final model by systematically removing the least significant covariates (Bolker et al., 2009; Bursac et al., 2008). The final model included driver’s age at citation, sex, citation before or after COVID-19 emergency declaration, and traffic offenses of speeding, no operator’s license, ACDA, seatbelt violations, and other offenses. Goodness-of-fit tests were conducted separately for both models. The likelihood ratio test yielded a chi-square value p > 0.995, indicating a strong fit between the reduced model and the data (Duchesne et al., 2010). Franklin County population estimates from the US Census Bureau were used to calculate rates per 10,000 population of ages 15–17.

## Results

Of the 5364 unique individuals included, 62.2% were male, 59.4% were 17 years old, 60.1% were White, and 86.1% were non-Hispanic (see Figure 2). Nearly 60% of citation tickets (59.4%) were issued before the COVID-19 emergency was declared. There were 9009 traffic offenses resulting from 6232 citation tickets (see Supplementary eTable1).

Of the 9009 traffic offenses, the most frequent were speeding (28.5%), no operator’s license (22.9%), and failure to control (6.9%) (see Table 1). Almost 20% (19.3%) of the traffic offenses were categorized as “Other”. 1.2% (n=111) of the total traffic offenses was for “Operating a Motor Vehicle Impaired” (OMVI) (see Supplementary eTable1). Speeding offenses were significantly more common among teens aged 16 and 17 (26.6% and 31.5%, respectively) compared to those aged 15 (8.9%) (p<0.01). However, there was no difference in speeding offenses by sex. Almost half (44.1%) of the traffic offenses among 15-year-olds were a no operator’s license offense. Males, compared to females, had a higher proportion of a no operator’s license (24.4% vs. 19.8%, p<0.01) offense. Seatbelt violations were more common among males (2.5%) than females (1.4%, p<0.01), but there was no significant difference across age groups. Black teens had more than twice the proportion of no operator’s license offenses than White teens (29.0% vs. 13.1%, p<0.01).

Of the 13 712 unique court charges resulting from 6232 citation tickets, 38.2% were adjudicated juvenile traffic offender (84.1% of tickets), 17.9% dismissal – Rule 29 (39.3% of tickets), and 3.7% suspended license (8.6% of tickets) (see Table 2). Teens aged 15 years had a significantly higher proportion of adjudicated traffic offenses (47.0%) compared to those aged 16 (37.8%) or 17 (37.9%) (p<0.01). The adjudicated juvenile traffic offender disposition was not significantly different by sex. A larger percent of 15-year-olds received a dismissal – rule 29 (22.6%) than those aged 16 (18.1%) or 17 (5.9%) (p<0.05).

Nearly 14% (13.9%) of the 6232 unique citation tickets were repeated citations for one individual. The results from the final model revealed that the likelihood of repeated citations increased by 3.61 (95% CI = 2.76–4.73) for each additional year of driver’s age (see Table 3). Males were 1.78 times (95% CI = 1.43–2.21) more likely than females to have a repeated citation. Speeding, no operator’s license, seatbelt violations, and other traffic violations were associated with higher odds of repeated citations, while an ACDA traffic offense was associated with lower odds of repeated citations. A traffic offense for not wearing a seatbelt was associated with the highest odds of a repeated citation (OR=8.22; 95% CI: 3.09–21.86), followed by speeding (OR=2.29; 95% CI = 1.77–2.96).

## Discussion

This study found that traffic violations were more common among juvenile males. The most common traffic offense was speeding, while the most frequent court charge was being an adjudicated juvenile traffic offender. The likelihood of receiving repeated citations increased with age. Not wearing a seatbelt was associated with the highest risk of repeated citations, followed by speeding. These findings contribute to limited research among teen drivers cited for traffic violation and suggest that citation tickets may not be isolated events but rather indicative of persistent risky driving behaviors. Our findings align with existing research, which shows that traffic offenders across all age groups are at a greater risk than non-offenders for repeated citations (Summala et al., 2014), as well as crashes, and crash-related injuries and fatalities (Ayuso et al., 2010; Factor, 2014; Goldenbeld et al., 2013).

Our results indicate that males had a higher proportion of citation tickets, and a greater likelihood of repeated citations compared to females. This is consistent with established research on sex-related differences in driving behaviors (Carnegie et al., 2009), revealing that male teen drivers are prone to risky driving behaviors, including speeding and traffic offense recidivism (Barr et al., 2015; Carnegie et al., 2009). Our study found that Black teens received more no operator’s license citations, aligning with previous research on financial barriers to licensure among minority youth (Elliott et al., 2008; Heck et al., 2008). Racial and sex-related disparities in traffic citations highlight the need for tailored interventions. Future research should evaluate programs and policy changes aimed at reducing financial barriers to licensure. Qualitative research exploring minority youth’s licensing experiences could also shed light on systemic obstacles and help shape more effective interventions.

Consistent with existing literature, we found that speeding is the most common risky driving behavior. Speeding is the leading cause of crashes and injury severity (Stiles et al., 2023), contributing to approximately one-third of all motor vehicle fatalities (National Highway Traffic Safety Administration, 2021). Our findings underscore the critical role of speeding in crash involvement among young drivers as previously established (Abdel-Aty et al., 2012; Williams et al., 2006). The high rate of speeding violations suggests a need to strengthen current education and training programs that emphasize safe driving practices. Targeted traffic safety programs should prioritize speeding, particularly among high-risk teen drivers. Future research should evaluate existing strategies and explore alternatives, such as graduated penalties or enhanced driver training, to reduce repeat offenses effectively.

### Limitations

This study has some limitations. First, the data were drawn from a single juvenile traffic court in Ohio, so the findings may not be generalizable to other courts or regions with different policies or demographic profiles. Second, our classification of one-time offenses was based on the first citation within the study period; some individuals may have had prior citations outside this timeframe, which could affect interpretation of the results. Third, limited data on race and ethnicity restricted the depth of analysis. Finally, the dataset lacked contextual details such as citation circumstances (e.g., location, time of day, etc.), access to driver education, and socioeconomic status, all of which may influence driving behaviors and citation risk.

## Conclusion

This study offers valuable insights into patterns of traffic violation and recidivism among teen drivers, a population at elevated risk for MVCs yet often overlooked in research. Our findings indicate that males and those cited for speeding are more likely to receive repeated citations. Efforts to reduce these behaviors could play a significant role in improving traffic safety for teen drivers, underscoring the need for targeted, evidence-based interventions. By identifying high-risk groups and behaviors, our results can inform the development of comprehensive prevention strategies that address both individual behavior and broader systemic factors. Future research should examine contextual and behavioral factors contributing to these patterns to better guide intervention efforts aimed at reducing MVCs and associated injuries and fatalities among teen drivers.

## Supplementary Material

1

## Figures and Tables

**Figure 1.**
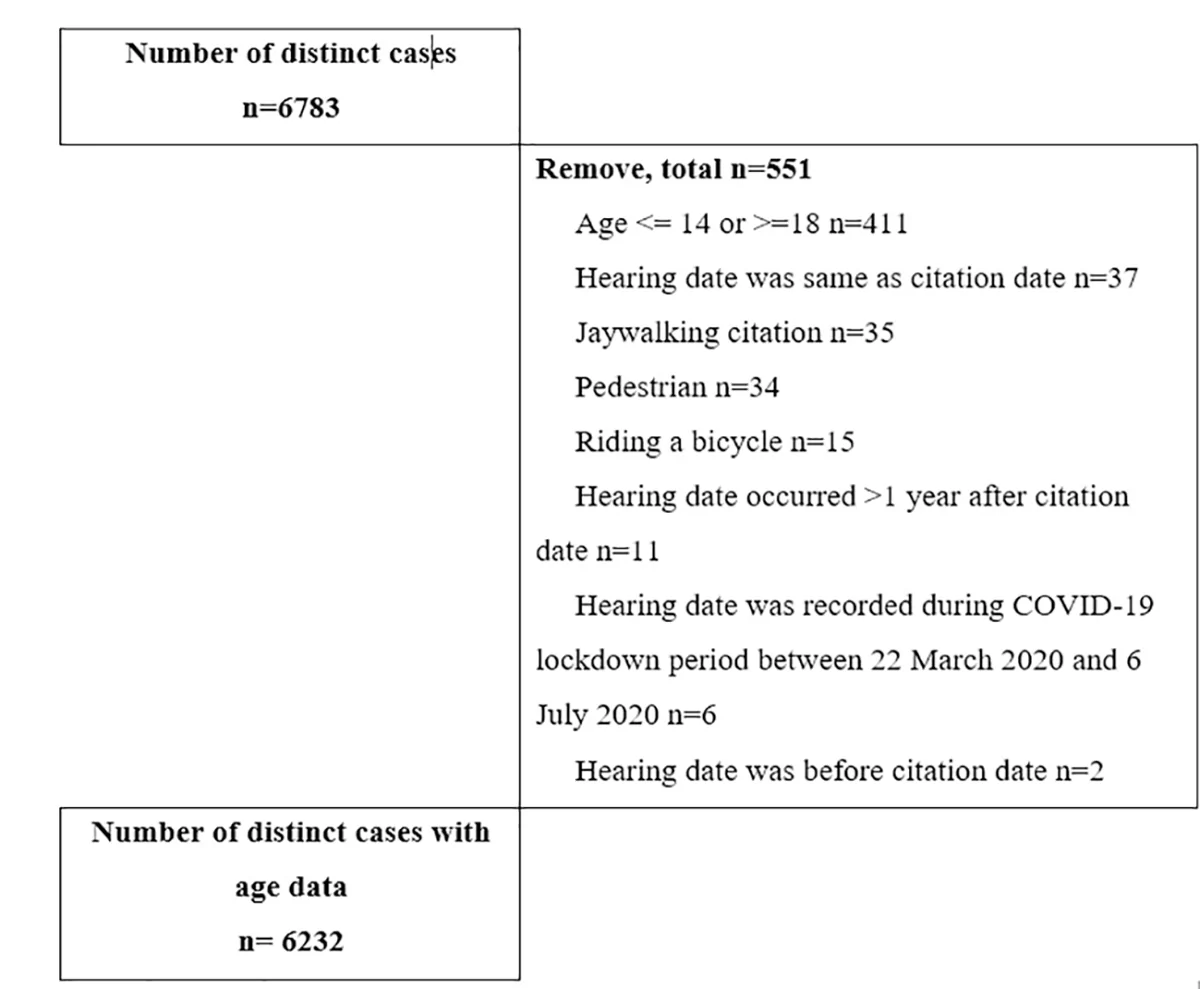
Flow Diagram

**Figure 2.**
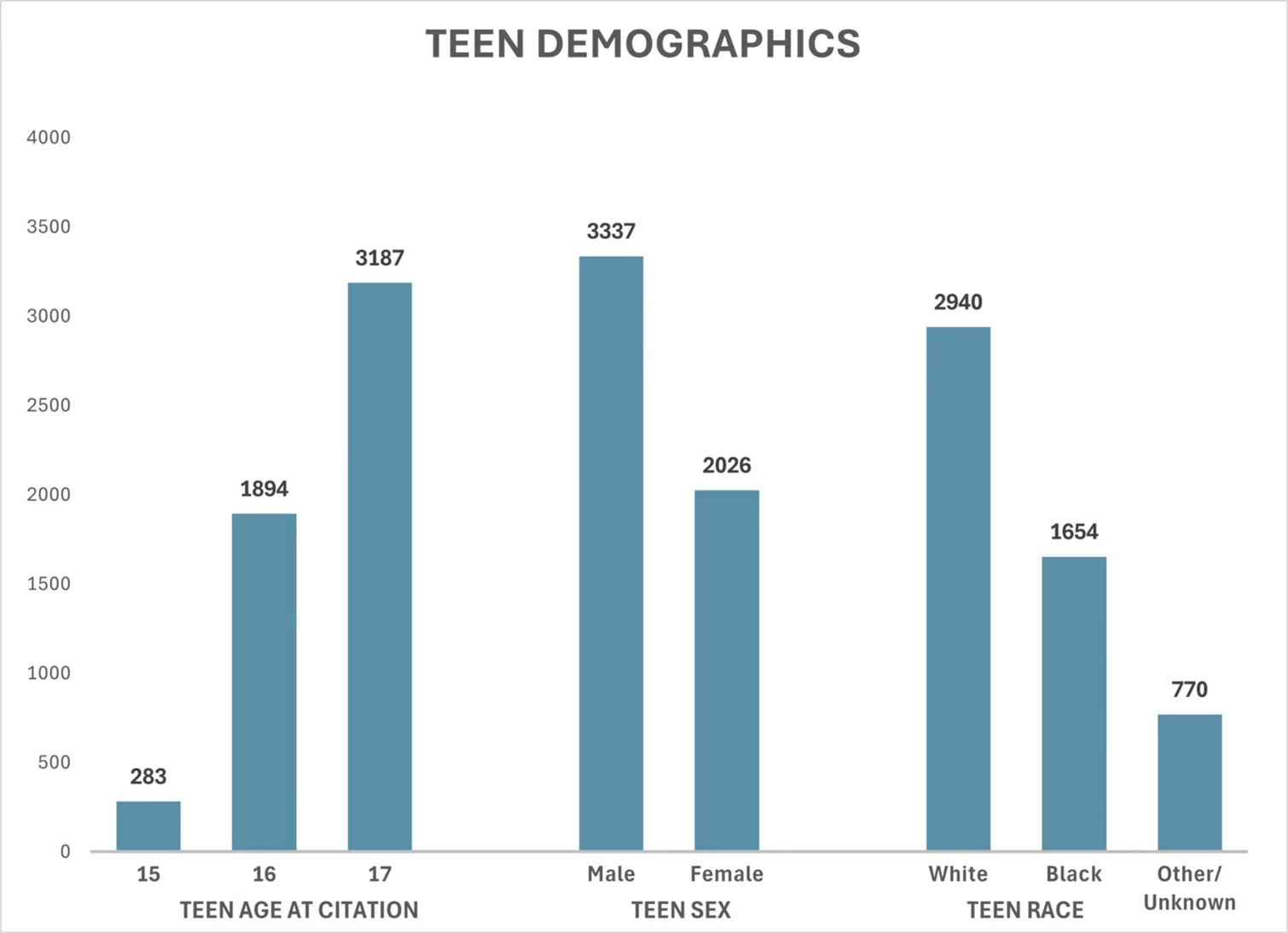
Teen Demographics (n=5364 unique individuals*) *Total for teen sex does not equal 5364 due to missing values (n=1)

**Table 1.** Top traffic offenses, overall and stratified by age, sex, and race

					Age				Sex^†^				Race^†^					
	Overall		15 years		16 years		17 years		Male		Female		White		Black		Other^††^	
	n	%^‡^	n	%^‡^	n	%^‡^	n	%^‡^	n	%^‡^	n	%^‡^	n	%^‡^	n	%^‡^	n	%^‡^

**Traffic Offenses**																		
**Total, traffic offenses**	**9009**		**571**		**2922**		**5516**		**5990**		**3018**		**4260**		**3220**		**606**	
Speeding	2568	28.5	51	8.9	777	26.6	1740	31.5**	1696	28.3	872	28.9	1539	36.1	714	22.2	124	20.5**
No Operator’s License	2060	22.9	252	44.1	660	22.6	1148	20.8**	1463	24.4	597	19.8**	557	13.1	934	29.0	214	35.3**
Failure to Control	624	6.9	40	7.0	212	7.3	372	6.7	403	6.7	221	7.3	309	7.3	222	7.0	33	5.5
ACDA^§^	459	5.1	6	1.1	183	6.3	270	4.9**	274	4.6	185	6.1**	332	7.8	87	2.7	14	2.3**
Failure to Yield	389	4.3	21	3.7	153	5.2	215	3.9*	201	3.4	187	6.2**	190	4.5	137	4.3	19	3.1
Right of Way	306	3.4	9	1.6	127	4.3	170	3.1**	160	2.7	146	4.8**	205	4.8	53	1.7	20	3.3**
Traffic Control Device	257	2.8	15	2.6	99	3.4	143	2.6	152	2.5	105	3.5*	125	2.9	92	2.9	22	3.6
Seatbelt Violations	206	2.3	12	2.1	56	1.9	138	2.5	163	2.7	43	1.4**	82	1.9	104	3.2	10	1.7**
Reckless Operation	162	1.8	17	3.0	59	2.0	86	1.6*	132	2.2	30	1.0**	62	1.5	78	2.4	13	2.2**
Driving Outside Marked Lanes	123	1.4	9	1.6	34	1.2	80	1.5	74	1.2	49	1.6	68	1.6	38	1.2	5	0.8
Failure to Stop at Stop Sign	118	1.3	5	0.9	45	1.5	68	1.2	65	1.1	53	1.8**	73	1.7	26	0.8	7	1.2**
Other^‡‡^	1737	19.3	134	23.5	517	17.7	1086	19.7**	1207	20.2	530	17.6**	718	16.9	735	22.8	125	20.6**

P-values of <0.05 and <0.01 are represented by * and ** respectively.

† Traffic offenses with missing sex and race information (n=1 and n=923, respectively).

†† Other: Asian, Indian, Native American, and Other.

‡ These percentages represent the column percent.

§ ACDA: Assured Clear Distance Ahead

‡‡ Other: OMVI, hit & skip, failure to comply, driving problems, problems with license, non-moving violations, slow speed, hazard zone, U-turn, problems with signal, tags, plate, lights, and equipment, unknown, and miscellaneous, etc. (see Supplementary eTable1)

**Table 2.** Court charges, overall and stratified by age, sex, and race

			Age						Sex^†^				Race^†^					
	Overall		15 years		16 years		17 years		Male		Female		White		Black		Other^††^	
	n	%^‡^	n	%^‡^	n	%^‡^	n	%^‡^	n	%^‡^	n	%^‡^	n	%^‡^	n	%^‡^	n	%^‡^

**Total, court charges** ^‡‡^	**13**	**100.0**	**474**	**100.0**	**4602**	**100.0**	**8636**	**100.0**	**8587**	**100.0**	**5124**	**100.0**	**8019**	**100.0**	**3718**	**100.0**	**786**	**100.0**
	**712**																	
Adjudicated juvenile traffic offender	5238	38.2	223	47.0	1741	37.8	3274	37.9**	3311	38.6	1926	37.6	2904	36.2	1517	40.8	328	41.7**
Dismissal - Rule 29^§^	2449	17.9	107	22.6	832	18.1	1510	5.9*	1538	17.9	911	17.8	1369	17.1	749	20.1	115	14.6**
Fine^§^	1866	13.6	67	14.1	627	13.6	1172	13.6	1218	14.2	648	12.6*	1029	12.8	527	14.2	121	15.4*
Costs assessed^§^	1570	11.4	34	7.2	573	12.5	963	11.2**	884	10.3	686	13.4*	1052	13.1	307	8.3	84	10.7**
Defensive driving course^§^	1497	10.9	9	1.9	541	11.8	947	11.0**	924	10.8	573	11.2	988	12.3	377	10.1	58	7.4**
Suspended driver’s license^§^	538	3.9	6	1.3	137	3.0	395	4.6**	350	4.1	188	3.7	348	4.3	110	3.0	37	4.7**
Limited driving privileges^§^	366	2.7	0	0.0	84	1.8	282	3.3**	231	2.7	135	2.6	268	3.3	63	1.7	18	2.3**
Community service^§^	188	1.4	28	5.9	67	1.5	93	1.1**	131	1.5	57	1.1*	61	0.8	68	1.8	25	3.2**

P-values of <0.05 and <0.01 are represented by * and ** respectively.

† Court charges with missing sex and race information (n=1 and n=1189, respectively).

†† Other: Asian, Indian, Native American, and Other.

‡ These percentages represent the column percent.

‡‡ Total court charges are greater as a traffic offense could have more than one court charge.

§ These charges apply to adjudicated juvenile traffic offenders.

**Table 3.** Adjusted odds ratios from generalized linear mixed model for repeated citation (yes/no) (n=6231 citation tickets)

	Full Model^†^ OR (95% CI)	Final Model^‡^ OR (95% CI)

Age	3.36 (2.63–4.31)	3.61 (2.76–4.73)
Sex (female=referent)*	1.77 (1.43–2.19)	1.78 (1.43–2.21)
COVID-19 (after COVID-19=referent)	0.77 (0.63–0.94)	0.77 (0.62–0.94)
Speeding	2.13 (1.35–3.37)	2.29 (1.77–2.96)
No Operator’s License	1.33 (0.77–2.32)	1.45 (1.07–1.97)
ACDA	0.71 (0.54–0.93)	0.71 (0.56–0.91)
Seatbelt Violations	7.82 (2.68–22.84)	8.22 (3.09–21.86)
Other^††^	2.06 (1.17–3.63)	2.23 (1.58–3.14)
Race*		n/a^‡‡^
*Black vs White*	1.09 (0.88–1.34)	
*Other/Unknown vs White*	0.94 (0.64–1.37)	
Ethnicity (non-Hispanic/unknown=referent)*	1.06 (0.66–1.70)	n/a^‡‡^
Failure to Control	0.92 (0.48–1.77)	n/a^‡‡^
Failure to Yield	0.76 (0.35–1.64)	n/a^‡‡^
Right of Way	0.59 (0.26–1.32)	n/a^‡‡^
Traffic Control Device	1.32 (0.59–2.98)	n/a^‡‡^
Driving Outside Marked Lanes	1.60 (0.52–4.95)	n/a^‡‡^
Failure to Stop at Stop Sign	1.02 (0.34–3.06)	n/a^‡‡^

* Individuals with missing race (n=468) and ethnicity (n=2,141) were coded as “Other/Unknown” and “non-Hispanic/unknown”, respectively, and included in the full model; the individual with missing sex (n=1) was excluded.

Goodness-of-fit test: Chi-square = 0.4879 (df = 19, p > 0.995).

† The AIC and BIC for the full model were 4675.73 and 4814.06, respectively.

‡ The AIC and BIC for the final model were 4663.02 and 4728.89, respectively.

†† Other: OMVI, hit & skip, failure to comply, driving problems, problems with license, non-moving violations, slow speed, hazard zone, U-turn, problems with signal, tags, plate, lights, and equipment, unknown, and miscellaneous, etc. (see Supplementary eTable1).

‡‡ n/a: not applicable
