# Supplementary material for: Traffic Offenses, Court Charges, and Repeated Citations Among Teen Drivers With Traffic Violations
Source: Traffic Saf Res (Lund). Author manuscript; Available in PMC 2026 Nov 1. (PMC13723337; doi:10.55329/jwot3323)
Supplement: Supplementary file 1 [file NIHMS2206093-supplement-1.pdf]

Supplementary eTable1. Frequency and percentages of all traffic offenses (n=51 categories)

Traffic Offenses	n	%
**Total**	**9009**	**100.0**
Speeding	2568	28.50
No Operator's License	2060	22.87
Failure to Control	624	6.93
ACDA†	459	5.09
Failure to Yield	389	4.32
Right of Way	306	3.40
Traffic Control Device	257	2.85
Seatbelt Violations	206	2.29
Reckless Operation	162	1.80
Driving Outside Marked Lanes	123	1.37
Failure to Stop at Stop Sign	118	1.31
Signals	117	1.30
OMVI‡	111	1.23
Parking and stopping	108	1.20
Hit & skip	103	1.14
All other vehicle lights	77	0.85
Display of tags	77	0.85
Suspension	71	0.79
Equipment	66	0.73
Starting and backing vehicles	61	0.68
C C without safety	58	0.64
Turn at intersection	58	0.64
Taillights	55	0.61
Headlights	53	0.59
Unauthorized plates	53	0.59
Change lanes	48	0.53
Invalid statute	48	0.53
Fail to display plates	46	0.51
Driving problems	45	0.50
Financial suspension	44	0.49
Miscellaneous	42	0.47
Fail to stop - not stop sign	40	0.44
Failure to comply	40	0.44
Problem with license	40	0.44
Direction and roadway	31	0.34
Improper passing	31	0.34
Probationary license violation	31	0.34
Registration	31	0.34
Distracted driving	22	0.24
Non-moving violations	18	0.20
U-turn	17	0.19
Unknown	16	0.18
Seatbelt - passenger	14	0.16
Child restraint	13	0.14
Minor	12	0.13
False information	10	0.11

Hazard zone	10	0.11
Slow speed	10	0.11
Hand and arm signals	4	0.04
Improper vehicle operation	4	0.04
Obscured tag/plate	2	0.02

†ACDA: Assured Clear Distance Ahead;
‡OMVI: Operating a Motor Vehicle Impaired

This is an open-access article distributed under the terms of the Creative Commons Attribution 4.0 International License (CCBY-4.0). View this license's legal deed at http://creativecommons.org/licenses/by/4.0 and legal code at http://creativecommons.org/licenses/by/4.0/legalcode for more information.
